# Supplementary material for: GmIAA27 Encodes an AUX/IAA Protein Involved in Dwarfing and Multi-Branching in Soybean
Source: Int J Mol Sci. 2022 Aug 3;23(15):8643. doi: 10.3390/ijms23158643 (PMC9368862; doi:10.3390/ijms23158643)
Supplement: Supplementary file 1 [file ijms-23-08643-s001.zip › ijms-1814241-supplementary.pdf]

Supplementary Figures

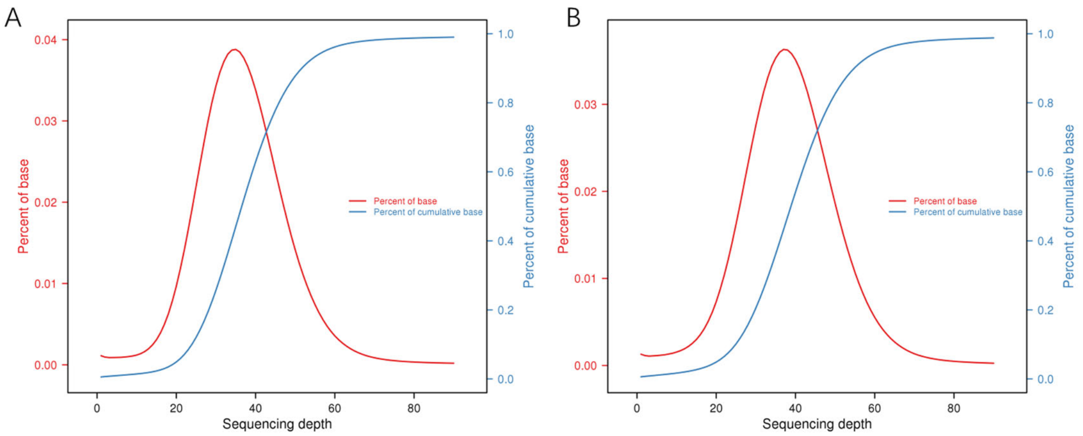

Figure S1 Sequencing depth distribution. (A) mutant type pool, (B) wild type pool. The abscissa is the sequencing depth, the left ordinate is the percentage of bases corresponding to this depth, corresponding to the red curve, and the right ordinate is the percentage of bases at and below this depth, corresponding to the blue curve.

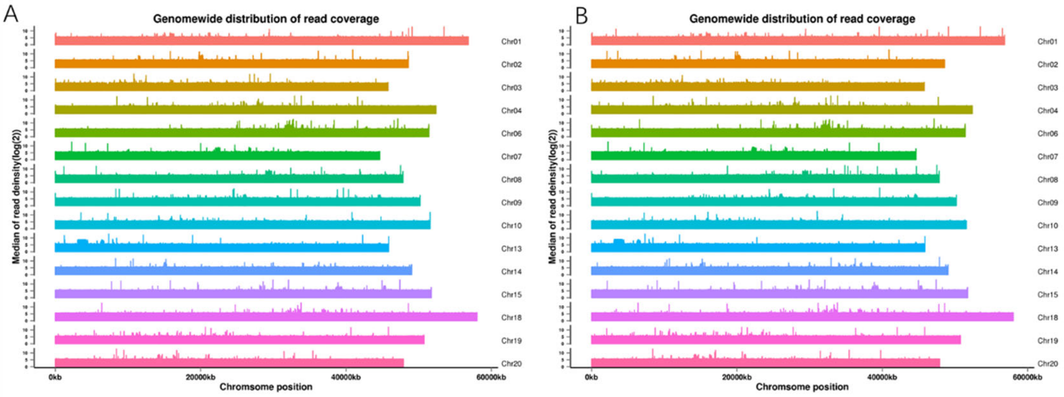

Figure S2 Chromosome coverage depth distribution map. (A) mutant type pool, (B) wild type pool. The abscissa is the position of the chromosome, and the ordinate is the value obtained by taking the logarithm (log_2) of the coverage depth of the corresponding position on the chromosome.

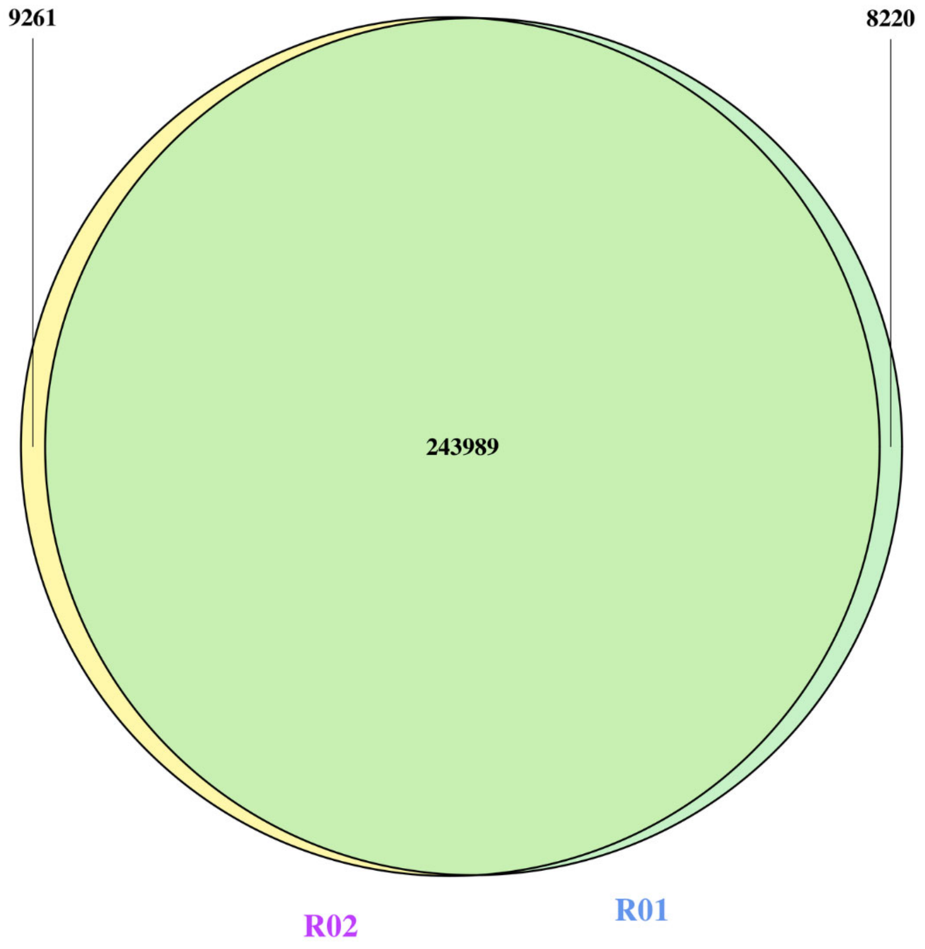

Figure S3 Venn plot The Venn statistics of the number of variant sites consider only whether the positions are the same and not whether the genotypes are the same. R01: mutant type pool; R02: wild type pool**.**

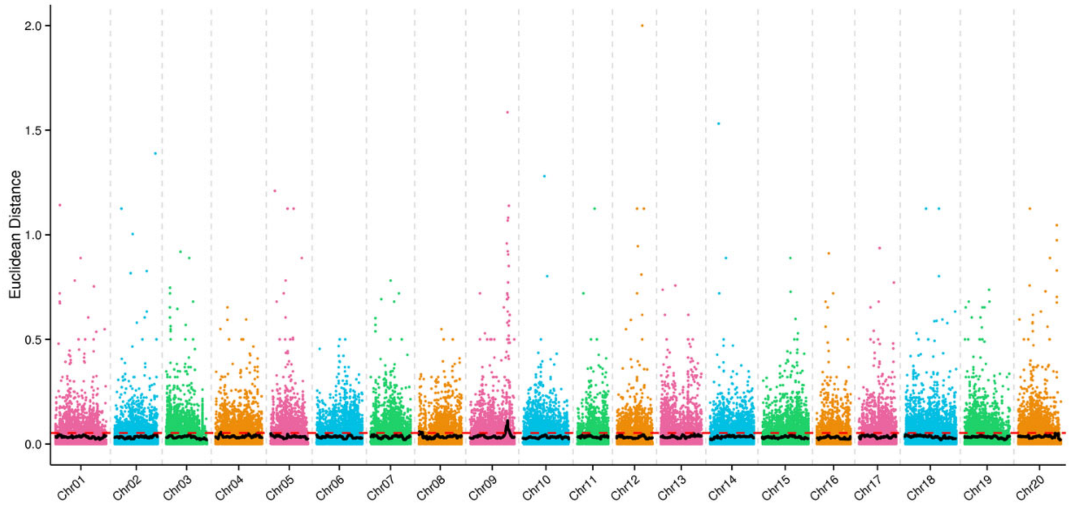

Figure S4 The distribution of ED association values on chromosomes. The abscissa shows the chromosome names, the colored dots represent the ED value of each SNP site, the black line the fitted ED value, and the

red dotted line the significant association threshold. The higher the ED value, the stronger the association effect of the point.

Supplementary Tables

ID	Clean reads	Clean bases	Q30 (%)	GC (%)
R01	132800306	39782627008	90.6	35.17
R02	144075679	43146730426	90.64	36.19

Table S1 The quality of resequencing data. ID: R01 was mutant type pool, R02 was wild type pool; Clean reads: the number of reads after filtering; Clean bases: the number of bases after filtering, the number of Clean reads multiplied by the sequence length; Q30 (%): the bases with a quality value greater than or equal to 30 The percentage of total bases; GC (%): the GC content of the sample; that is, the percentage of G and C t bases in total bases.

Chromosome ID	Start	End	Size (Mb)	Gene number
Chr04	47470000	47610000	0.14	25
Chr04	47810000	47980000	0.17	24
Chr04	5840000	5900000	0.06	14
Chr04	6040000	6150000	0.11	27
Chr08	2150000	3060000	0.91	194
Chr08	310000	2020000	1.71	370
Chr08	3550000	3640000	0.09	28
Chr08	4080000	4290000	0.21	68
Chr09	39780000	44850000	5.07	796
Total	-	-	-	1546

Table S2 Identification of target chromosome region by ED association analysis using SNP markers. Chromosome ID: chromosome number; Start: the starting position of the associated region; End: the ending position of the associated region; Size: the size of the associated region, in Mb; Gene number: the number of genes in the associated region.
